# A qualitative study to explore barriers to access behavioral health services among older people living with HIV in Georgia

**DOI:** 10.1080/09540121.2026.2618063

**Published:** 2026-01-22

**Authors:** Esma Imerlishvili, Maia Kajaia, Eka Chkhonia, Ramesh Raghavan, Mamuka Djibuti

**Affiliations:** a Partnership for Research and Action for Health, Tbilisi, Georgia;; b Faculty of Medicine, Ivane Javakhishvili Tbilisi State University, Tbilisi, Georgia;; c Department of Psychiatry, Tbilisi State Medical University, Tbilisi, Georgia;; d School of Social Work, New York University, New York, NY, USA

**Keywords:** Behavioral health, mental health, substance use, integrated care, HIV care, SDG 3: Good health and well-being

## Abstract

Older people living with HIV (PLWH) in Georgia experience a high rate of behavioral health disorders, the treatment for which requires an integrated approach. However, these older PLWH experience unmet needs for behavioral health services. We aimed to explore barriers for older PLWH to access behavioral healthcare services in Georgia. In 2024, we conducted 28 in-depth interviews with PLWH aged ≥40 years in four major Georgian cities. Participants were purposefully selected from a cross-sectional study sample based on their screened behavioral health disorders. We employed an inductive-deductive thematic analysis approach guided by the Ecological-Behavioral Framework for Healthcare Access and Navigation. Our study found that older PLWH face significant barriers to accessing mental health services, including different types of stigma, cultural beliefs about behavioral health disorders, mistrust in mental healthcare professionals, and a lack of patient-centered care. Fragmented healthcare systems also hindered access to care, particularly for those with substance use disorders. Social support and family responsibilities were key motivators for seeking help. A complex interplay of sociocultural, political, and individual factors hindered PLWHs’ access to behavioral health services. Addressing stigma through targeted interventions, raising awareness about behavioral health services, and constructing differentiated service models are crucial to improving access.

## Introduction

People living with HIV (PLWH) experience a higher rate of behavioral health disorders, such as depression, anxiety, substance – and alcohol-use disorders, compared to the general population (Olagunju et al., 2012; Rezaei et al., 2019). It has been estimated that 28−62% of PLWH have symptoms of one or more mental or substance use disorders (Lora et al., 2020). Behavioral health disorders among PLWH have been associated with poorer HIV treatment adherence, virological failure, diminished social and emotional well-being, and overall quality of life (Mayston et al., 2012; Mwangala et al., 2021). As the life expectancy of PLWH increases, they face a growing burden of behavioral health disorders, particularly in Low– and Middle-Income Countries (LMICs) (Olagunju et al., 2012; Rezaei et al., 2019) where access to appropriate healthcare is limited (Hoare et al., 2021; Remien et al., 2019).

Addressing these dual behavioral health and HIV needs is required to ensure both effective HIV care management and mental well-being of older PLWH (Razzouk et al., 2010). The importance and effectiveness of such an integrated approach to these two types of services is well-established in high-income countries (The UNAIDS Programme Coordinating Board, 2018; World Health Organization, 2016), though data remain scarce in LMICs (Nakimuli-Mpungu et al., 2021). Behavioral health interventions are complex, requiring an approach that addresses the diverse needs, socio-demographic characteristics, and cultural backgrounds of target populations, which are particularly varied among PLWH (Yang et al., 2024). Therefore, examining the local context and exploring the barriers and facilitators to implementing behavioral health interventions in resource-limited settings is needed.

Georgia is a low HIV prevalence country with a concentrated epidemic among key populations (UNAIDS, 2023), experiencing a high burden of stigma and discrimination (United Nations Population Fund [UNFPA], 2024). A cross-sectional study conducted in 2023 among older PLWH in Georgia revealed that this population has a high prevalence of behavioral health disorders as measured on standardized screening tests (Imerlishvili et al., 2025). However, to the best of our knowledge, there is no literature on the barriers to, and facilitators of, access to mental health services among this population in the country. The legacy of the Soviet Union’s institutionalized mental health system, which resulted in hospital-based mental health care and limited community-based and user-oriented services (Makhashvili & Van Voren, 2013), is also likely to impede access to mental health services among PLWH in Georgia.

To address this gap in the research literature, this qualitative study aimed to explore barriers for older PLWH to access behavioral healthcare services in Georgia. We aimed to inform the development of culturally relevant and effective mental health and substance use interventions and implementation strategies for older PLWH suffering from behavioral health disorders in Georgia.

## Methods

### Overall study design

This cross-sectional qualitative study employed in-depth interviews to explore perceived needs, barriers, and facilitators to accessing mental health and substance use services among older PLWH across urban and rural Georgia in 2024. We employed an inductive-deductive thematic analysis approach guided by the Ecological-Behavioral Framework for Healthcare Access and Navigation to identify individual and environmental factors facilitating and/or hindering access of PLWH to necessary behavioral health services (Ryvicker, 2018). We did not ask recipients about the treatment they received; instead, we focused on factors influencing the utilization of, and subjective experiences around, their care (as described in the Study Instruments section below).

The Ecological-Behavioral Framework for Healthcare Access and Navigation, developed by Miriam Ryvicker, synthesizes Andersen’s Behavioral Model of Health Service Use with the ecological model of aging to account for both individual – and environment-level determinants of healthcare use (Ryvicker, 2018). At its core, the framework emphasizes that healthcare navigation, defined as the dynamic processes by which individuals and caregivers navigate the healthcare system to identify, access, and utilize services, is shaped by the interaction of individual characteristics and contextual environments. Individual-level factors are categorized into predisposing, enabling, and need factors, which influence a person’s healthcare-seeking behavior and ability to navigate available services. These are complemented by provider factors, such as communication and trust, that shape navigation experiences and outcomes.

What distinguishes this framework is its integration of environmental domains, such as the healthcare environment (availability, affordability, and organization of services), the social environment (social support, stigma, and social capital), and the built environment (transportation, neighborhood characteristics, and physical infrastructure). Based on the framework, these domains act as moderators of healthcare navigation, influencing whether potential access translates into realized access. The framework, originally designed to examine the challenges faced by aging and vulnerable populations in the United States, has since been applied to research on health disparities, chronic disease management, and the barriers encountered by populations with complex health needs (Ryvicker, 2018). However, we considered its ecological orientation particularly useful for examining healthcare access in contexts like Georgia, where sociocultural stigma, fragmented service delivery, and variable infrastructure intersect to shape the behavioral health experiences of older PLWH.

### Study setting and population

In-depth interviews were conducted in the offices of community-based organizations (CBOs) working with PLWH, People Who Inject Drugs (PWID), and LGBTQI populations in four major cities: Tbilisi, Kutaisi, Zugdidi, and Batumi.

Our study participants were all ≥40-year-old PLWH who had participated in a cross-sectional quantitative study investigating cognitive and mental health disorders conducted in 2023 (Imerlishvili et al., 2025). In most literature, older people living with HIV (older PLWH) are commonly defined as individuals aged ≥50 years (Harris et al., 2018; McMillan et al., 2018). However, for this study, we included participants aged ≥40 years. This decision was made to address anticipated challenges in recruitment due to HIV-related stigma and to maximize participant involvement. By lowering the age cut-off, we ensured broader inclusion of eligible participants while still capturing a population experiencing age-related and behavioral health challenges.

Participants were selected based on the elevated scores they received on the following behavioral health screening tests administered in that study: (1) Drug use-related problems on Drug Use Disorder Identification Test (DUDIT): score ≥6 for men and ≥2 for women (DUDIT, 2026); (2) Alcohol use-related problems on Alcohol Use Disorder Identification Test (AUDIT): score of ≥8 (Scoring the AUDIT, 2026); (3) Mild-to-severe depression symptoms on the Beck Depression Inventory (BDI): score of ≥11 (Beck Depression Inventory (BDI), 2026); (4) Mild-to-severe anxiety symptoms on the General Anxiety Disorder-7 (GAD-7): score of ≥10 (Spitzer et al., 2006). To ensure a diverse range of experiences, we included participants of different sexes from various geographical regions.

### Study procedures

All eligible participants were contacted and invited to participate in the study. The recruitment started with those who scored highest on the behavioral health screening tests (DUDIT, AUDIT, BDI, GAD-7) and proceeded in descending order if someone refused to participate or could not be located. Ultimately, 28 participants were recruited before data saturation was achieved. During recruitment, four participants could not be located, and eight participants refused to participate for the following reasons: three had migrated to another country, one participant reported that her husband did not allow her to participate, and four participants declined because they did not want to talk about their experiences.

The face-to-face in-depth interviews between an interviewer and a participant lasted between 55 and 90 mins and were audio-recorded. Each participant was interviewed once for this study. Fieldwork took place from March to April 2024. The interviews were conducted by a female researcher (the first author), a PhD student in public health and epidemiology with prior experience in qualitative research and training in interview techniques and data analysis. The researcher had conducted a prior cross-sectional quantitative study, from which participants for this study were selected. As a result, the researcher had prior interactions with the study participants, which likely contributed to rapport-building during data collection.

### Study instruments and data analysis

We developed and used an in-depth interview guide to structure the interviews. It included questions and prompts on the following topics: (a) sociodemographic characteristics; (b) the knowledge, perception, and beliefs about mental health problems; (c) perceived mental health needs; (d) experiences of accessing mental health services; and (e) barriers and facilitators of access to these necessary services. The guide was iteratively refined based on initial feedback. We did not pilot-test the guide. However, its content was reviewed and validated by other authors who have expertise in qualitative research and public health. A professional transcriber was hired and audio recordings were transcribed. Since many participants did not have access to the internet or computer devices and lacked the necessary skills, transcripts and codes were not returned to participants for comment or correction. However, the interviewer ensured accuracy by carefully cross-referencing participant responses during the analysis process and discussing major themes and codes with the study team. The first author conducted coding using inductive-deductive approaches (Vanover et al., 2022). Instead of developing a coding tree, we manually coded the data and grouped the major themes in separate Excel sheets for easier organization and accessibility. The main themes were organized according to the domains of the Ecological-Behavioral Framework for Healthcare Access and Navigation (e.g., predisposing, enabling, and need factors; healthcare, social, and provider environments). Within each of these domains, however, codes were developed inductively based on participants’ narratives rather than being predefined. This combined approach ensured that our findings remained grounded in participants’ lived experiences while also being systematically organized in relation to a well-established conceptual framework. The entire process of data analysis was conducted in a Microsoft Excel spreadsheet.

Recognizing potential biases due to an interviewer’s (a female PhD student) academic background and personal interest in reducing health inequities, she used reflexivity practices, such as maintaining field notes to reflect on her assumptions critically. Additionally, to minimize bias during data analysis, codes and themes were discussed among the research team members to ensure a balanced interpretation of the data. The results of the data analysis are presented below in the results section of this article, and each theme is supported by a relevant quotation.

We presented major themes in the findings section, which are organized using headings and subheadings for clarity. In addition to the major themes, minor themes are also discussed to provide a more comprehensive understanding of the data. Quotations from participants representing these themes and cases are also included.

### Ethical considerations

Written informed consent was obtained from all participants before data collection. During this process, participants were informed about the study goals, the researcher’s academic background as a PhD student in public health and epidemiology, and the purpose of the research. The researcher’s role in conducting and analyzing the data was also explained. Participation was voluntary. Each participant was incentivized for their time and emotional resources (70 GEL, approximately 22 USD). The Institutional Review Board (IRB) at the National Center for Disease Control and Public Health in Georgia approved the protocol (IRB #2024-013).

## Results

A total of 28 PLWH (17 men and 11 women) participated in our study: 13 from Tbilisi, 6 from Kutaisi, 5 from Batumi, and 4 from Samegrelo. Their ages ranged from 41 to 70 years old, and they had been living with HIV for 2–25 years. All participants were receiving antiretroviral treatment (ART). A description of our sample according to mental health symptoms identified on screening tests is presented in Table 1.

### The main findings underpinned based on the Behavioral-Ecological Framework of healthcare access and navigation

Our study results below are grouped and presented according to the domains and subconstructs of the Behavioral-Ecological framework of healthcare access and navigation. Table 2 summarizes the main findings and includes Behavioral-Ecological Framework domains and themes that emerged during the data analysis.

### Individual characteristics

#### Predisposing factors.

The anticipated stigma associated with both HIV and mental health emerged as significant predisposing factors that prevent PLWH from accessing essential mental health services. Notably, the fear of HIV status disclosure and feeling of shame due to both HIV and mental health diagnoses create barriers not only to seeking mental health support but also to accessing other essential medical services. The stigma associated with mental disorder was more pronounced among family members of individuals with SUD.

“I didn’t tell her (the psychotherapist) my diagnosis. I told her my story as it was about someone else, someone who was abroad and needed advice. I hid it (my HIV diagnosis) there (from the psychotherapist) too”

– a woman from Tbilisi.

“I can’t go to the hospital when I need something. I’m ashamed that when they (medical personnel) take something (blood test), they will know (I am HIV positive) … ”

– a woman from Batumi.

“I was ashamed to visit a physician and to tell him that I had done something like this (a suicide attempt)”

– a woman from Tbilisi.

“I think only drug addicts would take such pills (psychoactive medications)”

– a woman from Batumi.

Cultural and historical beliefs about mental health and mental health professionals further hindered access to services. Participants often expressed mistrust of mental healthcare providers, preventing them from seeking help even when they recognized their need to do so. Moreover, some participants believed that mental health problems could be addressed through self-motivation and social support, rather than professional intervention.

“This problem (community belief), that psychiatrists and psychologists will drive you crazy, comes from the Soviet Union and continues to exist”

- a man from Tbilisi.

“I would always say, and I still think, that organic diseases are much better because there are some treatment options that can help you … Here, the management of these mental problems, I think, is impossible, unmanageable”

- a man from Tbilisi.

“I think so (medical help is not needed) because you should calm yourself down, a psychologist can’t help you”

- a woman from Tbilisi.

#### Enabling factors.

Financial constraints prevented some participants from accessing essential psychological services and purchasing necessary medication. Additionally, the lack of paid medical leave for self-employed individuals was identified as another barrier to accessing mental health services for some participants.

“I’m paid daily, so missing work means losing that day’s income,”

explained a man from Tbilisi.

“I’m supposed to visit the doctor every six months, but financial constraints have prevented me from doing so for two years. I’ve been continuously taking antidepressants during this time,”

shared a woman from Tbilisi.

Participants frequently reported facing long wait times for in-patient detoxification and neurology services. This hindered their access to timely care. Access to state-funded inpatient detoxification services was particularly challenging due to lengthy wait times. Moreover, long wait times for neurology services were associated with a shortage of qualified healthcare professionals.

“If you want to get qualified neurological services, you need to wait for months, because it is difficult to visit highly qualified professionals”

– described a woman from Tbilisi.

“If you pay, you don’t need to wait, but you have to wait a minimum of 3 months to get narcology services free of charge”

– a man from Kutaisi.

#### Need factors.

Despite experiencing anxiety and depressive symptoms, many individuals did not seek appropriate mental health services due to the aforementioned barriers. In contrast, individuals with alcohol and substance use disorders often failed to recognize their own mental health needs. Notably, individuals with SUD believed that dependence itself is not a mental health issue, but the behavior of those who use drugs. They reported that harmful behaviors that should be managed by mental health professionals are anger, aggression, violence, fear, etc.

“While drug use itself might not be a disorder, individuals struggling with substance abuse may require professional help. For instance, when substance use leads to harmful behaviors, such as family abuse,”

explained a participant from Zugdidi.

Additionally, problems with evaluating these needs appeared to hinder PLWH from accessing necessary mental healthcare. Specifically, none of our respondents reported an experience of being screened or assessed for mental health problems.

“They knew something serious had happened; the girl who left the AIDS center attempted suicide. They witnessed it, they knew who I was, they had my phone number, but nobody reached out to offer support or professional help”

– explained a woman from Tbilisi who had attempted suicide after being informed about her HIV diagnosis.

### Environmental factors

#### Healthcare environment.

Some participants, particularly those from Tbilisi, reported being offered psychotherapy and peer support services, typically through referrals by their doctors at the AIDS center. However, few participants from Tbilisi and most from other locations reported not being offered such services. Most who accessed the services expressed satisfaction, but some highlighted challenges. For example, psychotherapists at the sole PLWH community-based organization providing state-funded psychosocial services often developed personal relationships with community members through activities like training sessions, retreats, trips, and camps. Consequently, some participants felt uncomfortable sharing personal experiences with psychotherapists they viewed as friends.

“I visited her (a psychotherapist) twice and it helped me. Now, I again want to visit her, but I cannot. I am uncomfortable talking with her and sharing my personal experiences, because she is more than a psychotherapist”

– a woman from Tbilisi.

Participants also reported significant challenges navigating the healthcare environment to access mental health services. These difficulties were particularly severe for PLWH with substance use disorders. Challenges included difficulty identifying appropriate healthcare professionals and unclear pathways to receiving necessary mental health care.

“I had some symptoms and visited a neurologist, then a psychiatrist. However, they did not take my complaints seriously. Finally, I visited a narcologist and I have been taking his prescribed pills (for anxiety) ever since”

- a man from Tbilisi.

Our study also revealed a lack of integrated mental health services within the care provided for substance use disorders. Many participants either avoided or were dissatisfied with mental health services due to fragmented service delivery. Participants emphasized that psychological support, alongside medication, was essential for recovery.

“It didn’t help me, that’s exactly what psychology was needed for, I wasn’t mentally ready and I couldn’t do it alone physically (it [medication] does not help you)”

- a man from Kutaisi.

Additionally, the characteristics of inpatient healthcare settings for individuals with SUD created barriers for PLWH to complete their treatment. Participants perceived these settings as restrictive and non-patient-centered, describing them as prison-like environments.

“You’re up on the fourth floor, locked in. The window only opens this much (points with his hand to indicate a small opening). The rope is tied so that no one can escape. It’s like a prison”

- a man from Kutaisi.

#### Social environment.

Social environment emerged as a significant factor in choosing facilities for mental health support – preferences for where to access mental health services varied by place of residence. Specifically, the fear and shame of being identified as HIV-positive when seeking mental health services at the AIDS center were more pronounced among those living in small cities. In these communities, where people are more likely to know one another, being seen at the AIDS center could lead to assumptions about a person’s HIV status. In contrast, participants from Tbilisi preferred to access mental health services at the AIDS center, as the anonymity of a larger city reduced the likelihood of encountering acquaintances. Both groups believed their choice of setting would help minimize the risk of discrimination and stigmatization.

“If you’re admitted to the second floor of the AIDS center, everyone knows that you’re HIV-positive. Of course, it would be better if it (mental health services) was delivered separately (outside the AIDS center)”

- a man from Zugdidi.

“Yes, you’ll get (services) there (in the AIDS center) easily without many obstacles. They (psychotherapists) must know your HIV status and everything about you. So, it will be easier to get services there (in the AIDS center)”

– a man from Tbilisi.

The importance of social support in mental health was underscored by our findings. Participants living alone, particularly those with substance use disorders, frequently reported feelings of hopelessness and a lack of control over their future as significant barriers to accessing mental health services.

“I’m afraid of the future … I am afraid that there will be no other alternative in the future. I am unemployed, I do nothing. And then even if I stop using it, I will start using it again”

- a man from Zugdidi.

Conversely, study participants reported that responsibility for caring for their children and family members was the main motivation for them to access any, including mental healthcare services.

“I wanted it myself; I wanted to quit substance use because I thought about my son and grandchildren, and I didn’t want them to see the life I had. I didn’t want them to be excluded from the community because of me. That’s why I decided to quit substance use”

– a man from Kutaisi.

### Provider factors

Study findings revealed that provider-related factors, such as communication style, confidentiality practices, and attitudes towards patients with mental health concerns and/or SUD, can significantly hinder or promote PLWH’s access to and experience of mental health services. Participants reported instances of discriminatory behaviors, breaching confidentiality, and denying the provision of services from healthcare professionals, which created a barrier to seeking and receiving appropriate care.

“The doctor came out into the corridor while I was waiting with 3–4 other people. The doctor then asked, ‘Who is the HIV patient?’ Imagine, what I felt at that moment”

- a woman from Tbilisi.

“After she (the doctor) saw that the referring clinic was the AIDS center, she told me that her working day was over and she was unable to consult me”

– a woman from Tbilisi.

Participants reported ignorance or dismissive attitudes from healthcare professionals toward their behavior and complaints regarding mental and substance use disorders.

“They usually say, there is nothing to worry about (about mental health symptoms), calm down, don’t be afraid of, don’t be nervous”

- a woman from Zugdidi.

“I needed it (the pills) for sure. However, I couldn’t get a prescription, as we’re all tarred with the same brush … Doctors thought I was lying when I asked for medicine”

- a man from Zugdidi.

It should be mentioned that some participants expressed trust in their healthcare providers at the AIDS center, indicating that they would follow their advice to seek mental health services, even if they didn’t personally feel the need.

“I trust my doctor (at the AIDS center). If she recommended that I seek mental healthcare, I would go. However, she hasn’t. It makes me feel that I don’t need it”

- a man from Tbilisi.

### Health behavior and outcome

Our study found that a combination of HIV-related and broader societal stigma toward mental health hindered PLWH’s access to mental health services, often driving them toward self-medication.

“If I need something (medical help), I go to the pharmacy and ask them (based on the symptoms) for medicines to treat myself”

- a woman from Batumi.

Additionally, long wait times for essential services, such as in-patient detoxification at narcology centers, forced some participants to resort to self-care, even in critical situations.

“I think only drug addicts would take such pills. I am ashamed of being asked why I am taking these drugs and I don’t allow myself to go to a physician; Instead, I ask for medicine from pharmacists”

- a woman from Batumi.

“I had to wait ages or pay a fortune. So, I just decided to take care of it myself. My neighbor’s a nurse, so she gave me the infusion”

– a man from Kutaisi.

Despite the challenges, some participants found comfort and relief in social support. However, HIV-related stigma often led them to social isolation and prevented them from sharing their concerns with their immediate social circle.

“I stopped communicating with my friends and relatives. I thought from other people’s perspective. I was afraid that the finger would be pointed at me because of HIV stigma and the community’s belief that those who are infected have bad behavior”

- a woman from Tbilisi.

While participants did not report specific instances of health deterioration, they frequently expressed dissatisfaction with the quality of healthcare services, treatment discontinuation, and the lack of resolution for their health problems.

“I was there (visited a doctor) once and then gave up. It (treatment) was not what I needed; it (treatment) did not help me so, I stopped it (treatment)”

– a man from Zugdidi.

## Discussion

Our findings highlighted the complex interplay of sociocultural, structural, and individual factors influencing behavioral healthcare access among PLWH in Georgia. Specifically, HIV-related stigma, stigma associated with mental health issues, and the misconception that mental health problems can be self-managed emerged as key barriers that prevented participants from seeking essential support. Furthermore, low perceived need for care, especially among individuals with substance use disorders, and low evaluated need for mental and substance use disorders services among HIV care providers, were significant barriers to access. Finally, difficulties navigating the healthcare system – particularly among participants with substance use disorders – further compounded barriers to finding appropriate care and support.

The stigmas related to HIV, mental health, and substance use identified in our study appear to be deeply rooted in cultural norms and are manifested as internalized stigma (negative societal views internalized by individuals, leading to self-shame) (Earnshaw & Chaudoir, 2009), anticipated stigma (expectation of rejection or discrimination due to HIV status or mental health conditions) (Quinn & Chaudoir, 2009), and enacted (negative behaviors based on actual HIV status experienced in healthcare delivery settings) (Scambler, 2009) stigma. Addressing these forms of stigma requires comprehensive stigma-reduction interventions. In addition to such interventions, psychoeducation is essential to foster demand for appropriate care. The efficacy of psychoeducation among PLWH in improving mental healthcare has been demonstrated in other studies (Van Luenen et al., 2018). Based on our findings, educational efforts should target raising awareness among PLWH about recognizing mental health symptoms, an understanding that these symptoms are evidence for a possible disorder, and an appreciation of the importance of seeking professional support for these conditions. Furthermore, psychoeducation interventions should also help individuals with substance use disorders recognize that substance use leads to dependence – a condition necessitating professional intervention.

Regardless of the complexity of the behavioral healthcare access barriers among participants, our study revealed that most participants trust their physicians at the AIDS center, which could serve as a significant asset in facilitating behavioral healthcare access among older PLWH. This trust represents a foundational advantage, as it can encourage individuals to engage more readily with behavioral health services when referred by providers they trust. Physicians at AIDS centers are therefore uniquely positioned to bridge the gap between patients and needed mental health or substance use disorder services. However, our findings also highlighted that providers lack the knowledge to adequately identify the needs for behavioral healthcare and sometimes they lack skills to provide culturally competent care. Therefore, successful implementation will necessitate appropriate policy reforms, provider sensitization, and training to address institutionalized stigma and gaps in knowledge regarding behavioral health needs, as identified in our study. Furthermore, the development of standardized protocols to screen for behavioral health symptoms during routine HIV care visits, comprehensive training of staff when individuals screen positive, and regular supervision of healthcare staff to ensure that appropriate referrals are occurring is crucial to promoting timely, adequate, and sensitive mental health care, as also demonstrated in the literature (Moitra et al., 2024).

The emphasis on referral as a way to assure behavioral health access is based on the Georgian context. Integration of mental health screening and care services within HIV care settings has been demonstrated in other studies to improve both mental health and HIV-related outcomes (Razzouk et al., 2010). However, the integration of services for mental health and substance use disorders into HIV care may not always be practical or appropriate in the Georgian context. Our findings suggest that participants’ preferences for accessing mental health and substance use disorder services differ depending on the size and characteristics of their city. While co-locating and co-delivering behavioral health and HIV care may be possible in larger cities, individuals living in smaller towns appear to value the confidentiality that results when there is a separation between behavioral health and HIV treatments and providers. These variations highlight the importance of tailoring service delivery models to align with local needs and preferences.

Given the dual priorities of service integration and maintaining confidentiality, the implementation of differentiated service delivery models may offer a more effective solution (Ehrenkranz et al., 2021). These models could provide flexible approaches, such as offering integrated services in larger urban settings while establishing standalone mental health and substance use care options in smaller communities. This differentiation would address the unique cultural, structural, and logistical challenges of service delivery and ensure that services remain accessible and confidential. Additionally, offering multiple settings where PLWH can access mental health services, including psychotherapy, could address participants’ concerns about the limited availability of providers offering psychological services covered under government funding.

Social capital and family responsibilities, particularly caring for children, appeared as strong motivators for PLWH to prioritize their mental health. However, as PLWH age, these motivators may diminish due to decreased family obligations. PLWH then may face a double burden – increased mental health needs and decreased motivation to seek care. This aligns with broader trends in aging populations, where individuals often experience increased isolation, mental health challenges, and healthcare needs (Aoki et al., 2018; Cacioppo et al., 2014; Chen & Schulz, 2016). Our participants did not explicitly raise these age-related challenges, likely because our study involved relatively young participants who may not fully represent the needs of older PLWH related to the above-mentioned issues. Moreover, in Georgia, strong social ties and family obligations often persist into older age, potentially mitigating some of these challenges.

Our findings should be interpreted in the light of the study limitations. Firstly, the results presented here may be more positive than the actual scenario for most PLWH in Georgia. Notably, this sample may have had greater access to various psychosocial services provided by community-based organizations than the general population of PLWH, as the initial sample for the 2023 pilot study was recruited with the help of community-based organizations.

## Conclusion

The study revealed a significant gap between the behavioral health needs of PLWH in Georgia and the lack of availability of integrated services to address them. Major barriers to accessing necessary behavioral health care included HIV – and mental health-associated stigma, a lack of trust in mental healthcare professionals, and the absence of a standardized approach to addressing behavioral health problems within HIV care settings. Therefore, to improve access, enhancing the quality of care within HIV clinics, developing targeted antistigma and psychoeducational interventions, and creating differentiated service programs is necessary to ensure that PLWH with behavioral health needs are able to access high-quality care.

## Figures and Tables

**Table 1.** Number of participants according to mental health symptoms and cities.

A Qualitative Study to Explore Barriers to Access Behavioral Health Services among Older People Living with HIV in Georgia								
	SUD		AUD		Depression		Anxiety	
Tbilisi	2	2	4			3	1	3
Kutaisi	4		1				2	
Batumi					1	3	1	1
Zugdidi	1		2		2	1	2	

Men; Women.

**Table 2.** Matrix summary of the behavioral-ecological framework of healthcare access and navigation.

A Qualitative Study to Explore Barriers to Access Behavioral Health Services among Older People Living with HIV in Georgia	
**Individual Characteristics**	
Predisposing factors	HIV-associated stigma Mental health-associated stigma Mistrust in mental health professionals Misperceptions about mental health problems
Enabling factors	Financial barrier Employment policy Long wait period
Needs factors	Low perceived need Low evaluated need
**Environmental Characteristics**	
Healthcare environment	Fragmented mental health care Lack of high-quality care Lack of patient-centered care
Social environment	Cultural stigma Social support Responsibility in caring for family members
**Provider Characteristics**	Inappropriate clinical communication HIV-associated discrimination Relationship building
**Health Behavior and Outcome**	Self-medication Discontinuation of treatment Self-isolation Unresolved medical problems

## Data Availability

Original data collected within this study are not publicly available, as they might contain sensitive information.
